# A novel machine learning-based prediction method for patients at risk of developing depressive symptoms using a small data

**DOI:** 10.1371/journal.pone.0303889

**Published:** 2024-05-22

**Authors:** Minyoung Yun, Minjeong Jeon, Heyoung Yang

**Affiliations:** 1 Center for R&D Investment and Strategy Research, Korea Institute of Science and Technology Information, Seoul, Korea; 2 École nationale supérieure d’Arts et Métiers, Paris, France; 3 School of Education & Information Studies, University of California, Los Angeles, Los Angeles, LA, United States of America; 4 Center for Future Technology Analysis, Korea Institute of Science and Technology Information, Seoul, Korea; BRAC Business School, BRAC University, BANGLADESH

## Abstract

The prediction of depression is a crucial area of research which makes it one of the top priorities in mental health research as it enables early intervention and can lead to higher success rates in treatment. Self-reported feelings by patients represent a valuable biomarker for predicting depression as they can be expressed in a lower-dimensional network form, offering an advantage in visualizing the interactive characteristics of depression-related feelings. Furthermore, the network form of data expresses high-dimensional data in a compact form, making the data easy to use as input for the machine learning processes. In this study, we applied the graph convolutional network (GCN) algorithm, an effective machine learning tool for handling network data, to predict depression-prone patients using the network form of self-reported log data as the input. We took a data augmentation step to expand the initially small dataset and fed the resulting data into the GCN algorithm, which achieved a high level of accuracy from 86–97% and an F1 (harmonic mean of precision and recall) score of 0.83–0.94 through three experimental cases. In these cases, the ratio of depressive cases varied, and high accuracy and F1 scores were observed in all three cases. This study not only demonstrates the potential for predicting depression-prone patients using self-reported logs as a biomarker in advance, but also shows promise in handling small data sets in the prediction, which is critical given the challenge of obtaining large datasets for biomarker research. The combination of self-reported logs and the GCN algorithm is a promising approach for predicting depression and warrants further investigation.

## Introduction

Depression is a serious social and medical problem that has intensified since the onset of the COVID-19 pandemic [1, 2]. Many people are experiencing what has been referred to as the “COVID blues,” making the situation even more concerning. In the fields of psychiatry and psychopathology, numerous studies have been conducted to better understand depression and develop effective prevention strategies [3–7]. One key area of investigation has been to gain insight into depression by examining factors that contribute to its development. One noteworthy approach is the network method introduced by Borsboom et al. in 2008 [8–12]. This approach provides a novel way to understand the interconnectedness of the various symptoms associated with depression. In 2013, Bringmann et al. introduced a network approach to analyze clinical data collected using the experience sampling methodology (ESM), a self-report-based data-gathering method [9]. A total of 129 participants self-reported 6 feelings (three negative and three positive) on a 7-point Likert scale 10 times over 12 days. Using log data, Bringmann identified interactions among the 6 feelings using multilevel vector autoregression (VAR), which was presented within a network structure that included details about the strength and direction of these connections. This innovative network approach provides a comprehensive understanding of the complex relationships between depression-related feelings. By examining the feelings that affect each other and how they interact, this approach offers insights into the structural properties of depression. In addition to providing a comprehensive understanding, the network approach offers the added benefit of presenting complex information in compact data form. Such data can be easily processed using machine learning algorithms, allowing for further analysis and classification.

In addition to understanding depression, another important area of investigation is prevention. Strategies to prevent the onset of depressive symptoms involve early detection, accurate diagnosis, and effective outcome prediction. To achieve this, it is important to detect both current and potential patients with depression to ensure a comprehensive cycle of care that encompasses these important elements. The diagnosis and prediction of depression commonly involve the use of biomarkers that have been actively researched. However, a clear and dominant biomarker for depression treatment has yet to be identified, and ongoing research is being conducted to identify diverse biomarkers [13]. Traditional biomarkers, such as neuroimaging and hormone levels, have been extensively studied, but they are not always easy to obtain because they require the patient to physically visit a research center or hospital [14, 15]. Advancements in technology, such as machine learning and digital devices, have led to the exploration of digital biomarkers of depression. Biomarkers such as physical activity and smartphone usage are being studied as potential indicators of depressive symptoms or being at risk of depression. However, privacy concerns pose a challenge for these biomarkers. Hence, collecting a substantial quantity of either traditional or digital biomarkers is a significant challenge. Without access to biomarker data, diagnosis, especially prediction, is challenging. Previous prediction research has achieved high accuracy rates but often relies on large volumes of data collected over extended periods [7, 16, 17]. The central question then arises: Can accurate predictions be made using limited, small datasets given the challenges associated with data sampling? As such, it is vital to develop a method that enables prediction for potential patients, even when working with limited datasets.

In this study, we leveraged clinical ESM data previously used in Bringmann’s research, as a biomarker to develop a predictive model for individuals at risk of developing depressive symptoms in the near future, even when dealing with a limited dataset. The clinical dataset consisted of information from 129 patients collected over a span of 12 days. Our aim is to demonstrate the feasibility of accurate predictions using small amounts of clinical ESM data and the network approach introduced by Bringmann’s research. To facilitate the achievement of this goal, we implemented a data augmentation step and employed a machine learning method known as the graph convolutional network (GCN) for prediction [18–23]. The data augmentation step addresses the limitations posed by the small volume of the dataset [24–26]. A GCN, a type of neural network model, serves as an efficient tool for graph classification. In 2016, Boschloo et al. statistically analyzed the relationship between individual symptoms and the onset of depression, highlighting the importance of studying individual symptoms using the network approach [27]. Unlike their work, we focused on utilizing the network as a whole to build a predictive model for patients who may be at risk of depression. To the best of our knowledge, this study represents the first attempt to use network data as a whole to predict depression.

This novel approach offers the possibility of developing effective treatment approaches for depression, enabling early diagnosis and interventions to improve patient outcomes. Furthermore, this study demonstrates the potential of using deep learning tools with small datasets. The remainder of this paper is structured as follows: the methodology, results, discussion, and conclusion.

## Methodology

**Fig 1:**
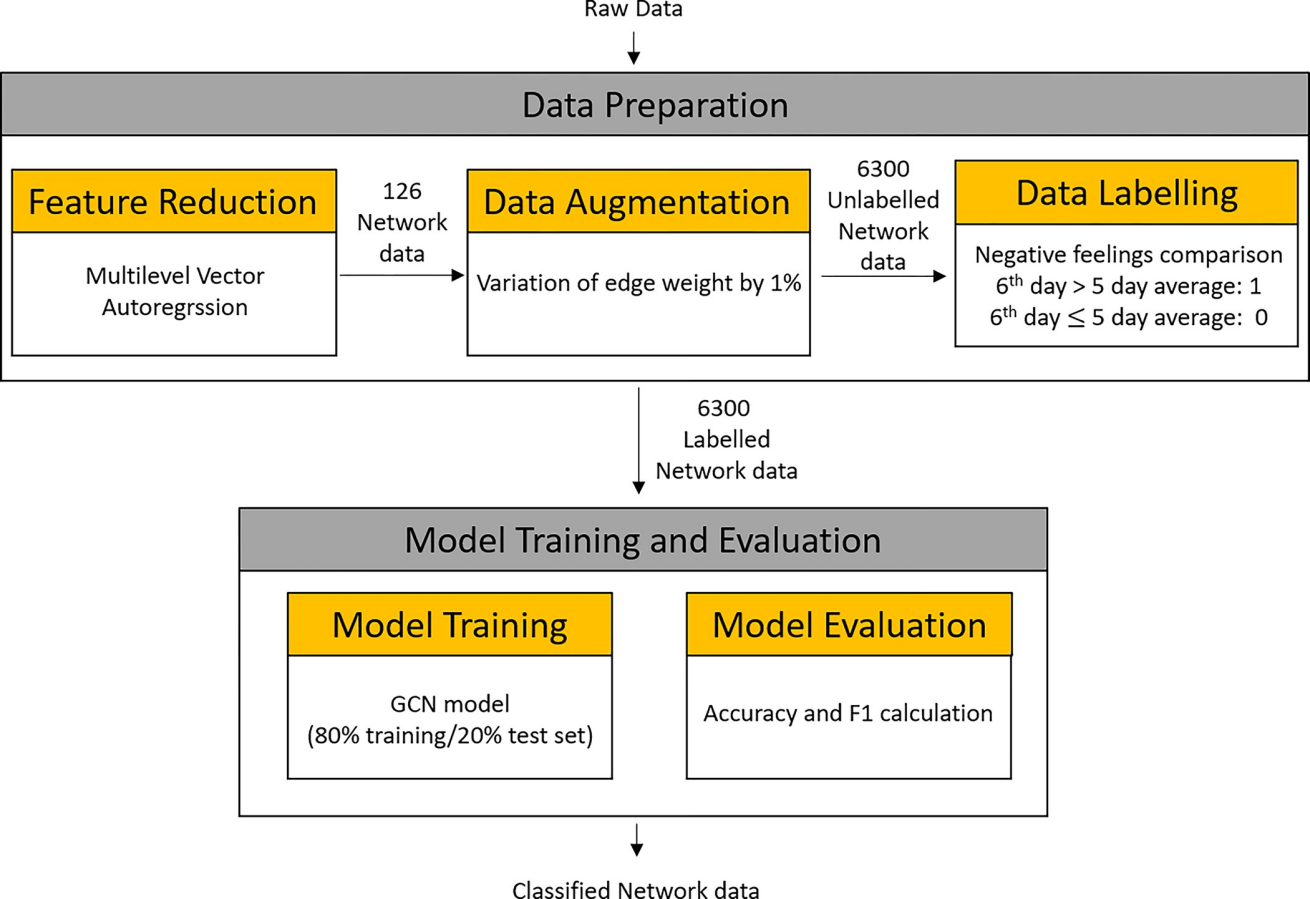
Schematic of the methodology with data preparation, model training, and evaluation.

**Fig 2:**
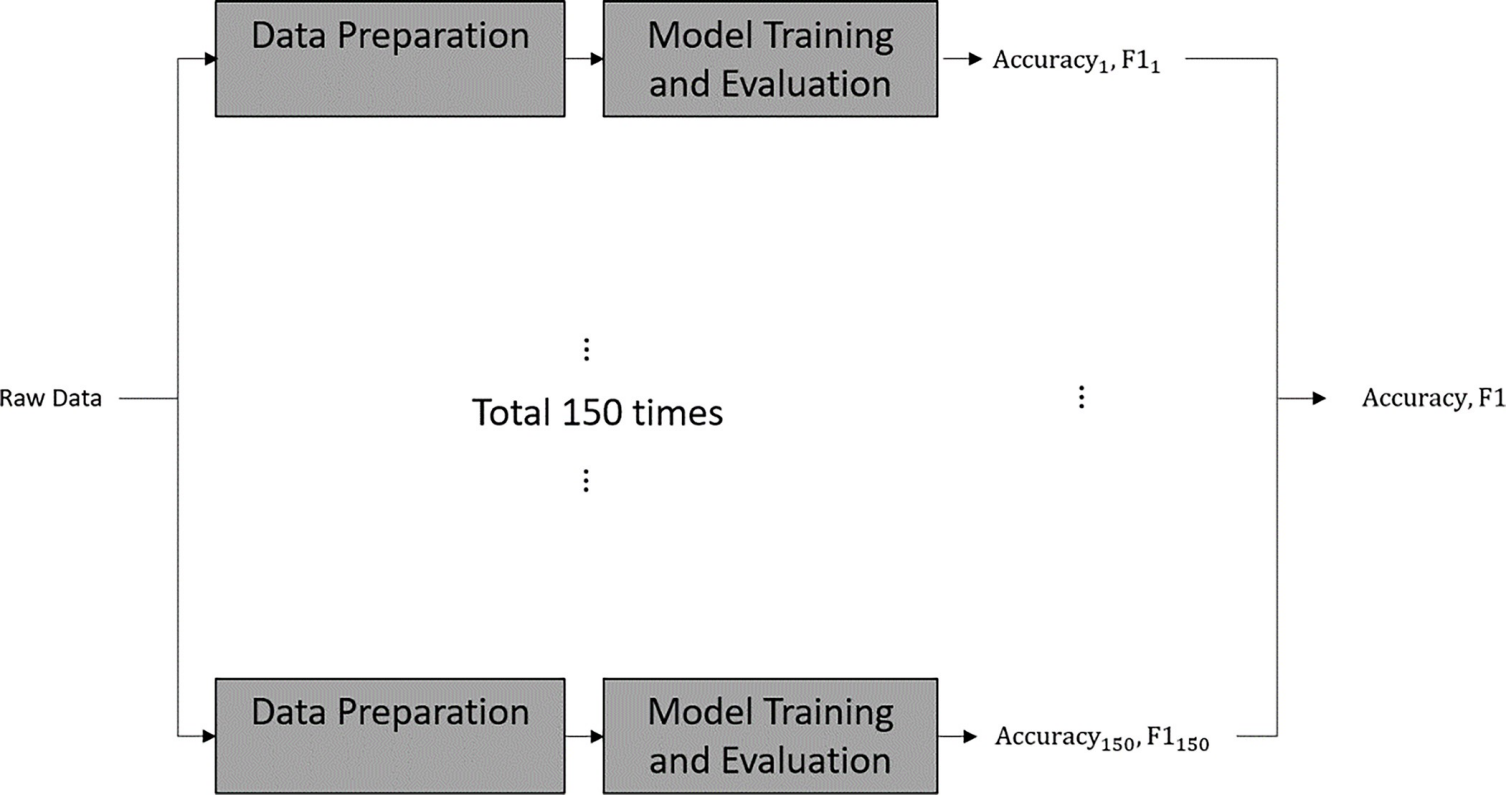
The repetition process to obtain 150 indicators.


The methodology consisted of two steps: (1) data preparation and (2) supervised model training and evaluation, as outlined in Fig 1. The data preparation steps include feature reduction, data augmentation, and data labeling. The prepared data then was used as input for supervised learning training during model training and evaluation. The indicators used in the evaluation step were accuracy and F1 (harmonic mean of precision and recall). To ensure the generalization of the model, we repeated the steps of data augmentation, model training, and validation 150 times to yield 150 indicators, which were averaged into the accuracy and F1, as displayed in Eqs 1 and 2. Fig 2 illustrates this process.

$$F1=\frac{\sum_{n=1}^{150}F1_{n}}{150}$$ Eq 1

$$Accuracy=\frac{\sum_{n=1}^{150}Accuracy_{n}}{150}$$ Eq 2

### Data preparation: Original data and feature reduction

We aimed to develop a predictive model for identifying depression-prone patients using the data provided by Bringmann et al., whose research provides two types of data: time-series data of 6 feelings recorded by 129 patients over 12 days, and R code for constructing individual networks from the raw data. Both can be accessed via the PLOS One website. The log data comprised clinical measurement data collected via self-reports of the 6 feelings (angry, fearful, sad, pleasantness of an event, cheerful, and relaxed) rated on a 7-point Likert scale. Participants were randomly prompted to self-report 10 times a day from 7:30 a.m. to 10:30 p.m. A total of 129 participants who had previously experienced depressive symptoms were divided into two groups: a control group and a treatment group undergoing mindfulness therapy. The treatment group comprised 63 participants with a mean age of 44.6 years (SD = 9.7), 79% of whom were female. In this group, 62% were employed either full-time or part-time, and 19% received illness or unemployment benefits. Additionally, 64% of the participants lived with a partner or family member(s). Notably, 56% had experienced more than three previous episodes of major depressive disorder (MDD) and 35% were simultaneously dealing with comorbid anxiety disorders at the time of sampling (51% before). The control group consisted of 66 participants with a mean age of 43.2 years (SD = 9.5), of whom were 73% female; 68% of the control group members were employed full- or part-time, while 23% received illness or unemployment benefits. Of the control group, 64% were living with a partner or family member(s); 45% had experienced more than three previous episodes of MDD, and 49% had comorbid anxiety disorders at the time of sampling (64% before). The sampling procedure, performed by Geshwind et al., was approved by the Medical Ethics Committee of Maastricht University Medical Centre. All participants signed an informed consent form [28]. For this study, we used data from 126 participants after excluding 3 participants with insufficient data points. Out of the 12 days of data collection, we only used the log data gathered during the first 6 days of the baseline study, whereas we did not include the log data from the following 6 days, which pertained to the evaluation of the effectiveness of depression treatment.

We used the first 6 days of log data from 126 participants to create a network for each patient. The process of converting the raw time-series data of the six feelings logged by patients into a simpler form (i.e., networks) is a type of feature reduction. The raw log data contained 10 markers per day for 5 days for the 126 patients (resulting in 50 data points per patient), which we reduced to 6 × 6 matrix forms (resulting in 36 data points per patient) using the network approach. Following Bringmann et al., we employed multilevel VAR to construct networks for each patient using data from the first 5 days of the 6 available. We used data points on the sixth day for labeling. Multilevel VAR, as presented by Bringmann et al., combines VAR and multilevel modeling to effectively handle both between-subjects and within-subjects clinical information [9]. The term “between-subjects” refers to variations observed among different patients, while “within-subjects” pertains to variations observed within an individual patient. This innovative method effectively addresses the complexities associated with nested longitudinal data.

### Data augmentation

The initial sample size of 126 was considered insufficient for the deep learning model of the GCN, highlighting the need to address the issue of small data. To overcome this challenge, we applied a sample augmentation step to increase the sample size. The goal of the augmentation was to generate data similar to the original sample but with a deviation of no more than 1%. To vary the edge values of an individual network, a uniform distribution with a minimum of -1% and a maximum of +1% of the original value was drawn using the Numpy library, a scientific computing package in Python. (The Python code can be found in the S1 Appendix.) A value is randomly chosen from this distribution and added to the original edge value. The edge values in a network represent the direction and strength of mutual interactions between depression-related feelings. As a result of the augmentation process, the augmented sample size of 6,300, 50 times larger than the original size, was created. The created networks with varied edge values (within 1%) were used as inputs for the deep learning model. The model accuracy and F1 were evaluated for the original data with and without augmented data.

### Data labelling

For supervised deep learning, three negative feelings (worry, sadness, and anger) were used for labeling because we aimed to predict which patients would exhibit more negative feelings in the future. Specifically, the study was interested in identifying participants who scored higher on these negative feelings on the sixth day compared to the average of the first 5 days. The participants who scored higher than the first five-day average was labelled as 1, while the others were labelled as 0 for binary classification. To test the efficiency of the model, three case studies were devised and are shown in Table 1. The cases varied in the ratio of negative feelings on the sixth day over the first 5-day average, allowing for binary classification with varying ratios of 1 to 0. The case studies are important as they assist researchers and clinicians in identifying patients who may develop more severe depressive symptoms in advance. However, when it comes to data and machine learning algorithms, training with a very small subset of data groups can lead to reduced learning efficiency. Given the current data distribution, cases with a ratio of the intensification of depression-related feelings (IDF) >1.2 account for only 28.5% of the total, while cases with IDF >1.5 account for only 12.6%. These limitations further complicate the training of predictive models for more severe depressive symptoms. Therefore, considering both the data distribution and IDF, cases with IDF >1.2 and >1.5 could serve as suitable criteria for case studies.

**Table 1:** Three cases with a varying ratio for GCN training.

Case	IDF	Proportion of potential depression cases (%)
1	>1	62.7
2	>1.2	28.5
3	>1.5	12.6

* Ratio of the intensification of depression-related feelings (IDF) = negative feelings on the sixth day/the first 5-day average. GCN = graph convolutional network

* Proportion of potential depression cases: percentage of patients labeled as depression-prone.

### Supervised model training and evaluation

Graph neural networks (GNNs), which are designed for classifying arbitrarily structured graphs, are efficient for supervised learning using graph data. Among the various types of GNNs, this study adopted the GCN for classification, which is widely used due to its ability to efficiently capture the local properties of graphs. For deep learning, the sigmoid function was used as the activation function; the optimizer was RMSprop with a learning rate of 0.001. The input for the GCN was composed of the labeled networks generated in the data preparation step, with nodes assigning the average value of negative feelings from the first 5 days for each participant, and edge weights representing interactive strengths among the feelings. The model was trained and tested using k-fold cross-validation with k = 5; that is, 80% of the sample was used for training and 20% for testing. The entire process, which involved data augmentation, model training, and validation, was repeated 150 times, as shown in Fig 2. The accuracy and F1 scores obtained from each run were used in Eqs 1 and 2 to calculate the final accuracy and F1 score, respectively.

## Results and discussion

The results of the trained model with the original data with the augmented data, including accuracy and F1 score, are presented in Table 2. Both indicators were calculated using the entire dataset (original data with augmented data, 6,300 pieces of network data). The accuracy and F1 score were around 86–92% and 0.83–0.90, which is deemed adequate. The high accuracy and F1 score indicate that the 1% variation introduced during data augmentation did not significantly affect the distinctiveness of the network edge values for individual cases, thus ensuring that the training process for predictions would remain minimally affected. The accuracy and F1 score for high intensification of depression-related feelings (IDF; 1.2 and 1.5) were high, signifying that this method would be effective at identifying patients who are likely to develop severe negative feelings in the near future.

**Table 2:** Accuracy and F1 for the three cases with the augmented dataset.

Case	IDF	Proportion of potential depression cases	Accuracy (%)	F1
1	>1	62.7	86	0.90
2	>1.2	28.5	87	0.83
3	>1.5	12.6	92	0.87

Classification became more difficult as the ratio of patients who scored higher on negative feelings on the sixth day compared to the first 5-day average increased (cases 2 and 3), as fewer samples are labeled as 1. This explains why the accuracy rose as the F1 score declined slightly as the majority of the samples were labeled as 0, resulting in a homogeneous pool.

Table 3 presents the results obtained with the original data from the 126 participants. The indicators obtained using the original dataset were as accurate as those obtained using the augmented dataset. The purpose of this case study with only original data was to confirm that the trained model would be appropriate for both cases, with or without augmented data. In addition, the accuracy increased with a higher IDF ratio because the original data was mostly labeled 0. The F1 score was > 0.90 in all three cases, suggesting that the model was robust.

**Table 3:** Accuracy and F1 for the three cases with the original dataset.

Case	IDF	Proportion of potential depression cases (%)	Accuracy (%)	F1
1	>1	62.7	89	0.94
2	>1.2	28.5	93	0.90
3	>1.5	12.6	97	0.91

## Conclusion

Depression is a serious social issue that has been studied using various methods for better understanding and prevention. Among these methods, a novel network approach has been developed to represent depression-related feelings using a compact network format. This network approach has the advantage of extracting essential data and eliminating superfluous information, making it ideal for further analysis. Our goal was to utilize small amounts of data in network form to identify patients with potential depression. We developed a novel algorithm that combines the network data of depression-related feelings with a deep learning method, GCN, to predict the likelihood of post-depression patients developing negative symptoms in a short timeframe. The issue of a small sample size was addressed using data augmentation, which boosted the sample size to 50 times the original size before model training. The validation process of the trained model resulted in accuracy and F1 scores above 80%, ranging from 86–97% for accuracy and 0.83–0.94 for the F1 score, which we deemed adequate compared to prediction performances of 70–95% by previous research [7, 17]. The contribution of this study is the development of a highly efficient predictive model using a limited amount of clinical data. This is in contrast to previous studies, which often relied on extensive datasets collected over extended periods ranging from one year to several years.

This method enables early detection and intervention, leading to more efficient depression-related treatments. Successful demonstration of this method yields important findings. First, network data showing the mutual interactions among relevant feelings is a good biomarker and input for further analysis, such as prediction or classification. Second, the issue of limited data in psychiatry can be addressed using data augmentation, which is crucial given the challenge of acquiring large datasets in the field. Third, this method has the potential for a range of applications in prediction or classification in the field of psychiatry.

The limitations of this study lie in the nature of the dataset used for the prediction. The prediction model could be enhanced with access to a more diverse dataset, particularly data from individuals who have not undergone treatment for depression. Furthermore, we relied solely on self-reported mood rating data, specifically on three negative feelings (sadness, anger, and fear) as biomarkers. Incorporating other types of depression-related biomarkers may enhance the predictability of the algorithm. Additionally, this study is based on a single case study design, and future research involving multiple case studies could further validate the method.

## Supporting information

S1 Appendix Supplementary code. (DOCX)
