# Supplementary material for: A novel machine learning-based prediction method for patients at risk of developing depressive symptoms using a small data
Source: PLoS One. 2024 May 22;19(5):e0303889. doi: 10.1371/journal.pone.0303889 (PMC11111038; doi:10.1371/journal.pone.0303889)
Supplement: S1 Appendix — (DOCX) [file pone.0303889.s001.docx]

**Appendix. Supplementary Code**

Import numpy as np

a_doclist = []

variation_percentage = 0.01 # 1%

for ns in range(50): #50 times larger sample size

# Generate random variations within the range of -1% to +1%

random_variations = np.random.uniform(-variation_percentage, variation_percentage, size=original_data.shape)

# Apply the random variations to the original data

varied_data = original_data * (1 + random_variations)

a_doclist.append(varied_data)
